# Stress distributions of the short stem and the tapered wedge stem at different alignments: a finite element analysis study

**DOI:** 10.1186/s13018-022-03425-6

**Published:** 2022-12-09

**Authors:** Nobuhiro Kaku, Jonas A. Pramudita, Kansei Yamamoto, Tsuguaki Hosoyama, Hiroshi Tsumura

**Affiliations:** 1 Department of Orthopaedic Surgery, Faculty of Medicine, Oita University, 1-1 Idaigaoka Hasama-Machi, Yufu City, Oita 879-5593 Japan; 2 Department of Mechanical Engineering, College of Engineering, Nihon University, 1 Nakagawara, Tokusada, Tamura, Koriyama, Fukushima 963-8642 Japan

**Keywords:** Finite element analysis, Arthroplasty, Hip, Femur, Prosthesis

## Abstract

**Background:**

The mechanical effects of stem length reduction and stem alignment on the surrounding femur remain unknown. This study directly compared the stress distribution on the surrounding femur of existing tapered wedge stems and short stems and examined the properties of stress distribution at different stem alignments in three dimensions.

**Methods:**

Finite element analysis was conducted for standing and walking. The cementless stem was appropriately sized to ensure adequate contact with the medial cortical bone line that contours the medullary cavity. The stem neck axis was aligned with the femoral neck axis in the mid-position and placed in 2° of the varus and valgus, 3° of flexion and extension, and 10° and 40° of anteversion.

**Results:**

Regardless of stem length, the trend of stress distribution was similar. The short stem generated less stress around the stem than the tapered wedge stem. In the coronal plane, the effect of varus and valgus deflection was small. In the sagittal plane, the stress generated around the stem was higher in the extended position than in the flexed position. In the horizontal plane, the stress generated around the stem was higher when the stem anteversion was smaller.

**Conclusions:**

Depending on the design, short stems can reduce the stress on the surrounding bone, compared to a longer tapered wedge with similar stress distribution. Additionally, a short stem can reduce the effect of the varus position. Stems should be placed to achieve stable initial fixation while noting that stresses increase with extension and reduced anteversion.

## Background

In total hip arthroplasty (THA), various types of stems exist, each with its own original fixation style. Given that stiffness increases with an increase in the stem size, less stress is transferred to the proximal femur, which may be a factor for stress shielding that is a long-standing problem in cementless stems [1]. Studies using X-ray imaging indicate that a larger stem size corresponds to a higher incidence of stress shielding [2]. Shorter stems have advantages in physiological load transfer from the proximal femur; thus, a smaller stem size may reduce stress shielding [3–5]. The cementless tapered wedge stem can be fitted in a smaller region, compared to conventional fit and fill stems, which occupy a larger femoral bone marrow cavity and press-fit into the proximal medullary cavity of the femur to attain the initial fixation. Using a shorter stem may be useful because it is less invasive to the femoral bone marrow cavity, and it preserves more bone. Short stems have been developed especially for young people under 50 years of age [6, 7], although they are a challenging method for initial fixation strength. Some reports on the short stem indicate that the initial fixation strength is comparable [8], although the short stem may have a higher risk of initial fixation and postoperative fracture owing to its small contact area [9–11].

Several investigators have reported on the effects of shortening the stem length on the initial fixation and weight-bearing from a mechanical point of view by using a virtual prosthesis design [2, 7]. However, existing short stems should be compared with tapered wedge stems to find more realistic results on the effects of stem length reduction. To the best of our knowledge, no mechanical study has directly compared existing tapered wedge and short stems. Based on several reports [12, 13], short stems should have a shape with an appropriate fit and a high medullary cavity occupancy at the proximal femur to obtain reliable initial fixation. The PROFEMUR® Preserve (MicroPort Orthopedics, Arlington, TN, USA), a metaphyso-diaphyseal fixation device, has a high medullary cavity occupancy rate and provides good outcomes with an average (range) survival rate of 78 (53–87) months postoperatively [14]. Moreover, PROFEMUR® TL tapered wedge stems, which are relatively long, are also produced by the same manufacturer. The strain energy density (SED) is used in bone remodeling simulations as a mechanical parameter to control bone remodeling [15]; however, its distribution has not been presented in most previous hip prosthesis-related parameter studies. To understand the contribution of the mechanical parameters to the femur in detail, the analysis of different types of mechanical parameters such as von Mises stress, SED, or maximum principal stress would be necessary.

Installing the stem at an anteversion angle different from that of the femoral neck depends on factors such as different anteversion of the femoral neck or combined anteversion theory. In addition, the tapered wedge stem, which is often flat, has more freedom in the direction of flexion–extension and rotation than does the conventional fit and fill stem. The short stem is placed at the proximal femur, including the femoral neck, which has a relatively wide medullary cavity; therefore, it is easier to lean than the planned stem alignment. However, very few mechanical reports have analyzed the effects of installing such stems at different alignments [16]. Finite element method (FEM) analysis is frequently used in joint prosthesis research because it can simulate the trend of stress distribution under all conditions [17]. However, to our knowledge, no studies exist on the stress analysis of femurs with the same stem in different coronal, sagittal, and axial alignments. Therefore, the aim of this study was to directly compare stress distribution on the surrounding femur of existing tapered wedges and short stems and to compare the nature of the stress distribution at different stem alignments in three dimensions.

## Methods

### Ethical approval

The study was conducted ethically in accordance with the principles of the Declaration of Helsinki and was approved by the ethical committee of Oita university (approval number: 1605; approval date: October 18, 2019). Written informed consent was obtained from the patient whose clinical case and femur were used in creating the model.

### Analysis model

Finite element modeling and finite element analysis were conducted using HyperMesh (Altair Engineering Inc., Troy, MI, USA) and LS-DYNA R11.1 (Ansys Inc., Canonsburg, PA, USA), respectively. The three-dimensional (3D) shape data of the femur were obtained by extracting only the femur from the computed tomography (CT) image by using 3D modeling software RETOMO (BETA CAE Systems International AG, Root, Switzerland). The CT image was of the right femur of a 46-kg woman who underwent THA for osteonecrosis of the femoral head with a standard intramedullary shape on imaging evaluation. The coordinate system of the femur was based on Bergman’s coordinate system [18]. For the stem coordinate system, the intersection of the stem axis and neck axis was set as the origin. The *z*-axis was set as the stem axis, the *y*-axis was a straight line drawn from back to front through the origin, and the *x*-axis was a straight line orthogonal to the *y*- and *z*-axes through the origin. The femoral neck of the 3D finite element model was resected, and the stem was inserted into the resection surface of the femoral neck toward the diaphysis of the femur.

**Fig. 1:**
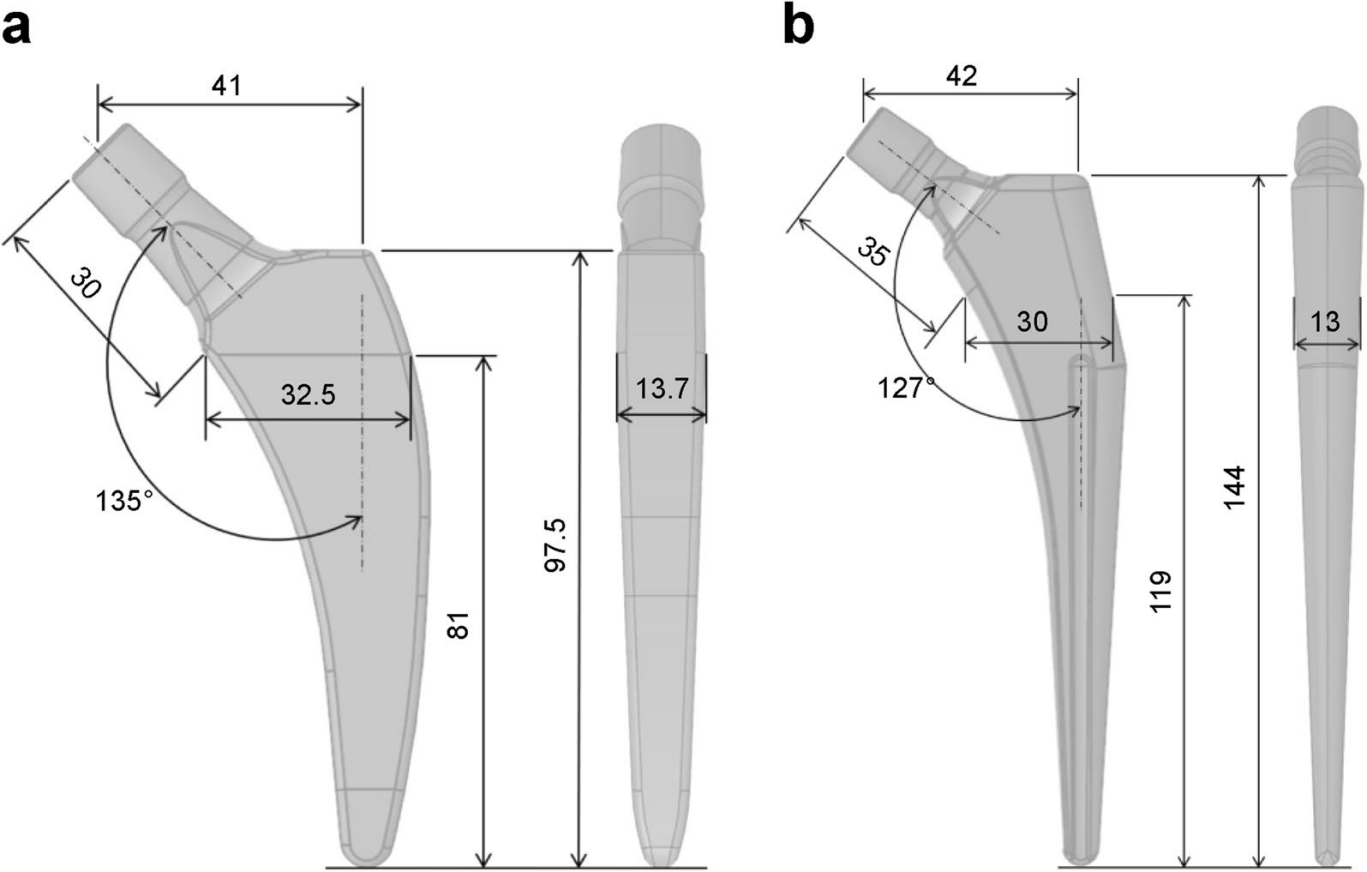
Dimensions of the short stem and the tapered wedge stem. **a** PROFEMUR® Preserve stem. **b** PROFEMUR® TL stem. The neck shaft angle is expressed in degrees (°) and the other measurements are expressed in millimeters (mm). PROFEMUR® Preserve and PROFEMUR® TL are manufactured by MicroPort Orthopedics (Arlington, TN, USA)

The computer-aided design models of the tapered wedge PROFEMUR® TL cementless stem (MicroPort Orthopedics, Arlington, TN, USA) and PROFEMUR® Preserve short stem (MicroPort Orthopedics) were used to determine the appropriate size of each stem with a CT-based simulation software (ZedHip Lexi Co., Ltd., Tokyo, Japan). The stem size was considered appropriate when the contour of the stem contacted the medial cortical bone line that outlines the medullary cavity. The dimensions of the short stem and the taper wedge stem are shown in Fig. 1. The femur and stem models comprised four-node tetrahedral elements with an element size of 2.0 mm. The mesh quality was evaluated by maintaining Jacobian and aspect ratio to be more than 0.7 and less than 5.0, respectively. The mesh size was decided by conducting a mesh sensitivity study with approximately 5% convergence error for the von Mises stress, based on previous reports [19, 20], resulting in an optimum mesh with element edge length of 2 mm. Furthermore, calculation time of less than 1 day was also taken into account for computational efficiency during the decision. The number of elements and nodes for each model is listed in Table 1. The meshed PROFEMUR® Preserve stem model had 4945 elements and 23,378 nodes, whereas the meshed PROFEMUR®TL stem model had 7864 elements and 40,040 nodes. In the stem installation of both models, the stems were installed at a stem height that did not cause any difference in leg length. Stem installation was based on the intermediate position where the stem axis coincided with the bony axis of the femur and the stem neck axis coincided with the femoral neck axis. The stems were installed at 2° for varus and valgus, 3° for flexion and extension, and 10° and 40° for stem anteversion. These conditions were used to guide the range that is likely to occur during the actual surgery. Excessive condition settings for stem alignment were avoided to pursue clinical reality.

**Table 1:** Nodes and elements of the FE models in the PROFEMUR® Preserve and PROFEMUR® TL stems

Stem position	PROFEMUR® Preserve				PROFEMUR® TL			
	Cortical bone		Cancellous bone		Cortical bone		Cancellous bone	
	Nodes	Elements	Nodes	Elements	Nodes	Elements	Nodes	Elements
Neutral	186,556	41,292	81,539	16,890	186,556	41,289	81,539	16,887
Flexion	192,267	42,537	83,633	17,208	192,267	42,534	83,633	17,205
Extension	193,070	42,636	80,841	16,710	193,070	42,633	80,841	16,707
Anteversion 10°	194,341	42,803	82,704	17,092	183,625	40,517	82,704	17,089
Anteversion 40°	188,776	41,916	83,189	17,151	188,776	41,913	83,189	17,148
Valgus	187,531	41,373	81,395	16,848	187,531	41,370	81,395	16,845
Varus	192,173	42,277	83,238	17,151	192,173	42,274	83,238	17,148

The PROFEMUR® Preserve is the short stem and the PROFEMUR® TL is the tapered wedge stem used in this study. PROFEMUR® Preserve and PROFEMUR® TL are manufactured by MicroPort Orthopedics (Arlington, TN, USA)

*FE* finite element

**Fig. 2:**
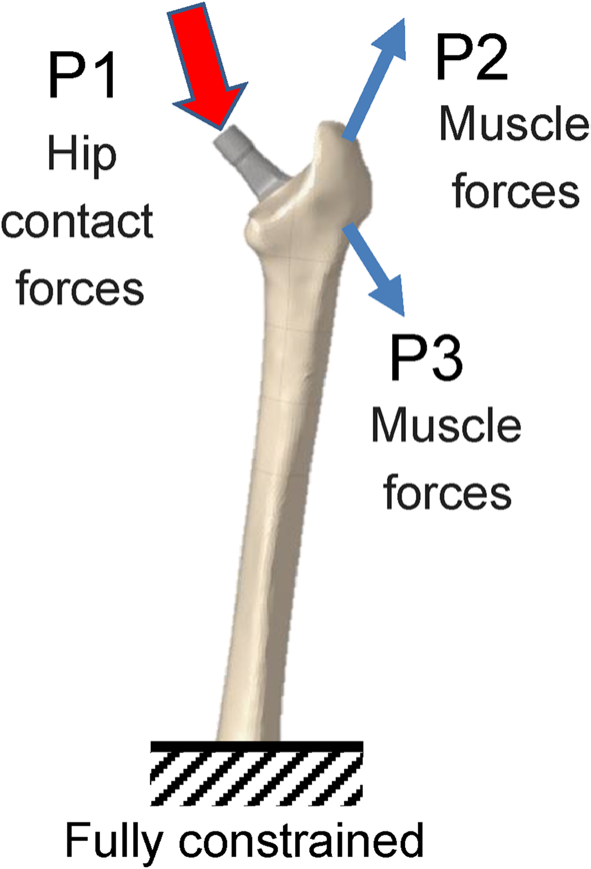
Forces and constraint applied to the finite estimate (FE) models during walking and standing simulations. Loading conditions for the postoperative femoral FE model are based on the report by Heller et al. using three action points (P) of the attachments or wrapping points of the muscles, which are labeled P1 to P3 [22]

**Fig. 3:**
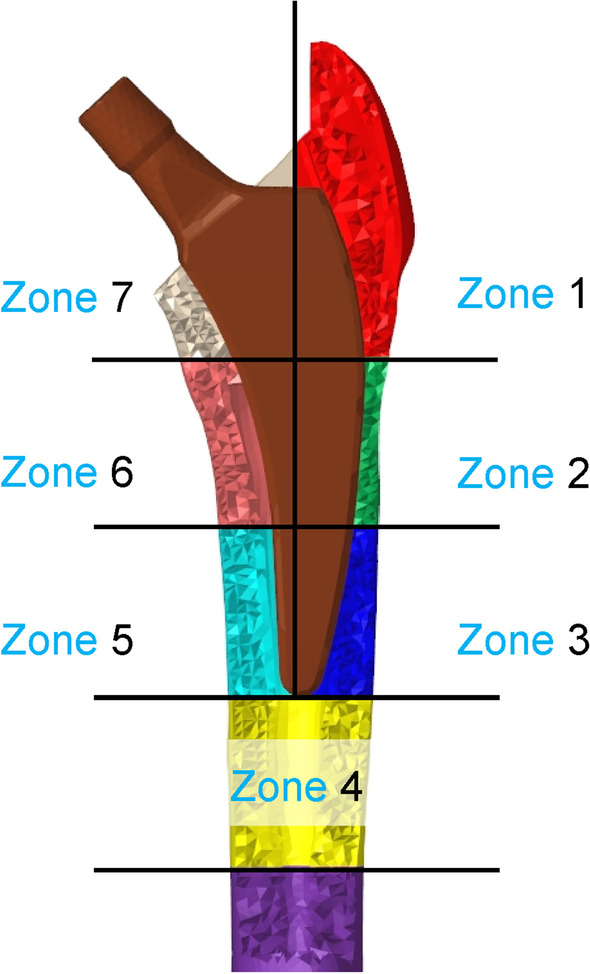
Seven volumes of interest (VOIs), based on the Gruen zone. The bone around the stem is divided into seven three-dimensional sections, based on Gruen zone classification

In the finite element analysis, the load and constraint conditions of the femur were set for two conditions: walking and standing. The distal femur was assumed to be fully constrained. The material properties of the finite element models and the loads acting on the femur and stem are shown in Tables 2, 3, and 4 and Fig. 2 [21, 22]. After the analysis, the volume of interest (VOI) was defined on the femur model at the contact point between the medial and lateral sides of the stem, based on Gruen zone (Fig. 3). However, the stem length between the two was originally different; therefore, the width of the zone set under the stem length was also different, as shown in Table 5. For the contact conditions between the stem and femur, the coefficient of static friction and the coefficient of dynamic friction between the stem and the cancellous bone, as well as the stem and the cortical bone, were set to 0.64 and 0.3, respectively [23]. The load was assumed to increase linearly and reach a maximum value at 0.2 s [24], and calculations were conducted using the dynamic implicit method. Mechanical parameters, including von Mises stress, SED, and the maximum principal stress in each VOI, were calculated. This study was conducted at Oita University (Yufu City, Japan) and Nihon University (Koriyama, Japan) from 2020 to 2021.

**Table 2:** Material properties (linear elastic materials) used in the finite element simulations

	Density (g/cm^3^)	Young’s modulus (GPa)	Poisson’s ratio
Cortical bone	1.80	17.5	0.3
Cancellous bone	0.80	10.0	0.3
Stem	4.43	113	0.3

**Table 3:** Load acting on the femur and stem during gait

	*X* (N)	*Y* (N)	*Z* (N)
P1	− 244	− 148	− 1034
P2	292	68.6	364
P3	− 4.1	83.5	− 419

The three action points (P) of the attachments or wrapping points of the muscles are labeled as P1, P2, and P3

**Table 4:** Load on the femur and stem in the standing position

	*X* (N)	*Y* (N)	*Z* (N)
P1	0	0	− 451.3

**Table 5:** Width of each zone and distance from the stem top edge of the FE models

	PROFEMUR® Preserve		PROFEMUR® TL	
	Zone width (mm)	Distance from the stem top edge (mm)	Zone width (mm)	Distance from the stem top edge (mm)
Zones 1 and 7	32.5	32.5	48.2	48.2
Zones 2 and 6	32.9	65.4	48.5	96.6
Zones 3 and 5	32.6	97.9	48.1	144.8
Zone 4	32.6	130.5	48.3	193.1

The PROFEMUR® Preserve is the short stem and the PROFEMUR® TL is the tapered wedge stem used in this study. PROFEMUR® Preserve and PROFEMUR® TL are manufactured by MicroPort Orthopedics (Arlington, TN, USA)

*FE* finite element

## Results

**Fig. 4:**
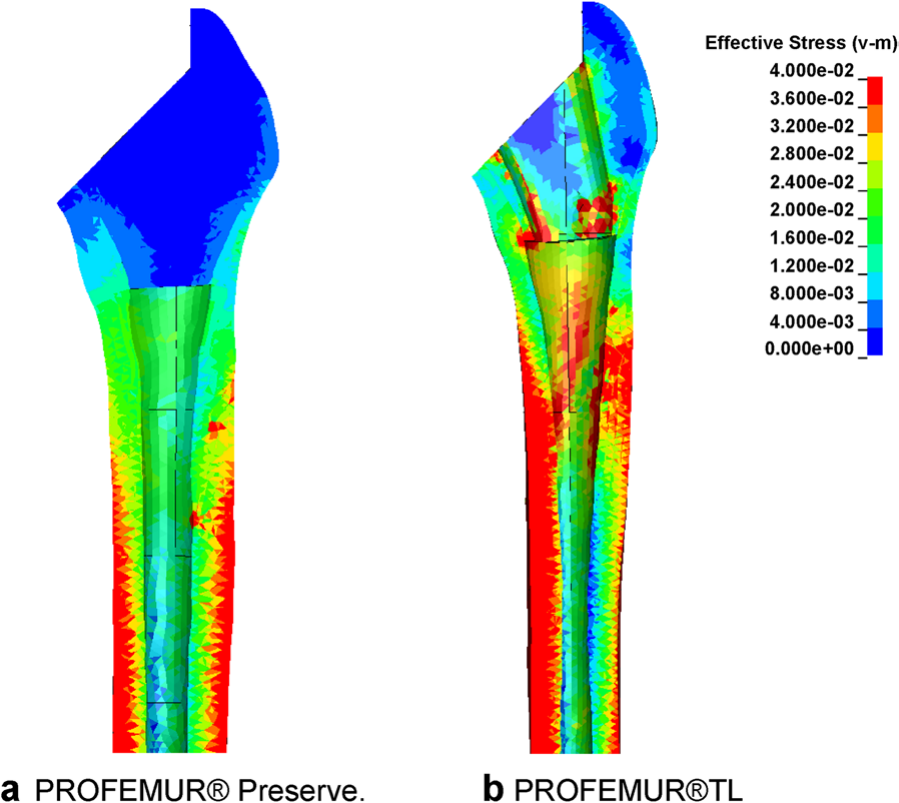
Von Mises stress distribution of femur models with neutral stem positions under waking condition. **a** PROFEMUR® Preserve. **b** PROFEMUR® TL. In the stress cloud maps, the areas of high stress were relatively fewer and confined to the distal portion of the stem in PROFEMUR® Preserve, compared to the PROFEMUR® TL. PROFEMUR® Preserve and PROFEMUR® TL are manufactured by MicroPort Orthopedics (Arlington, TN, USA)

**Fig. 5:**
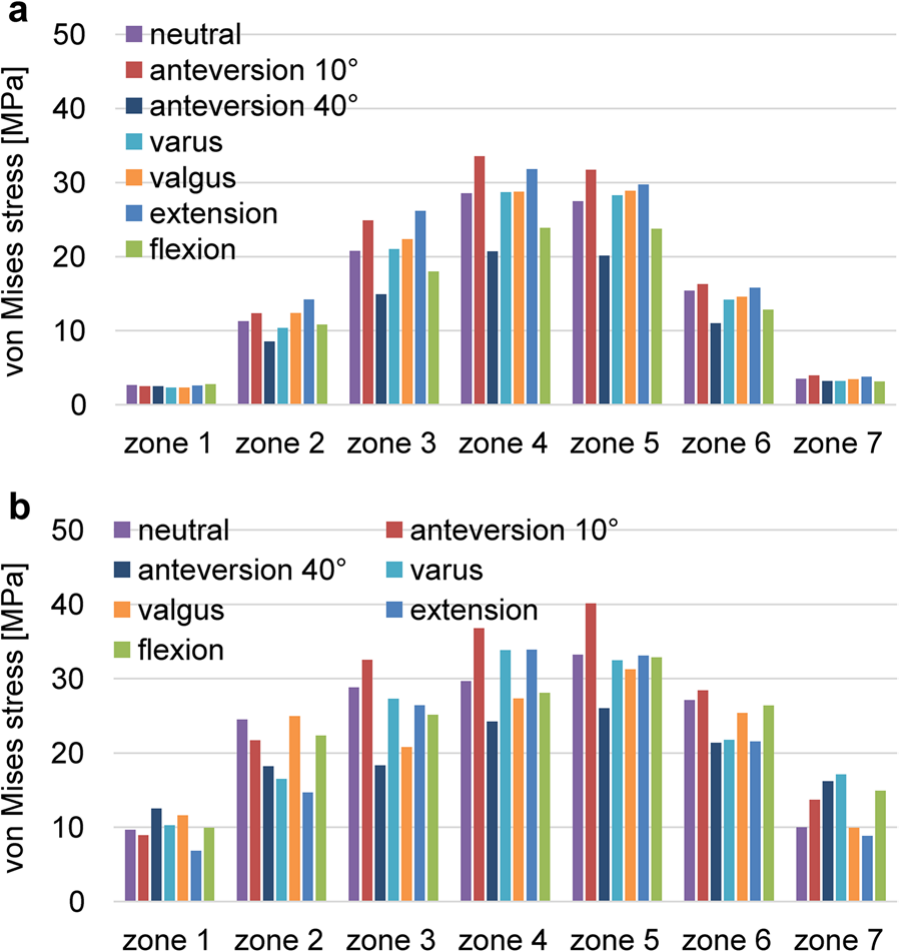
Comparison of the average values of von Mises stress under the walking condition. **a** PROFEMUR® Preserve. **b** PROFEMUR® TL. Von Mises stress during the gait condition shows a mountain-shaped von Mises stress distribution with zone 4 as the apex with the Preserve and zone 4 or 5 as the apex with the PROFEMUR® TL. In the PROFEMUR® Preserve, the von Mises stress generated around the stem is higher in the extended position than in the flexed position. The stem anteversion is different; however, the PROFEMUR® Preserve has lower stress in zones 1 and 7 than does the PROFEMUR® TL. PROFEMUR® Preserve and PROFEMUR® TL are manufactured by MicroPort Orthopedics (Arlington, TN, USA)

**Fig. 6:**
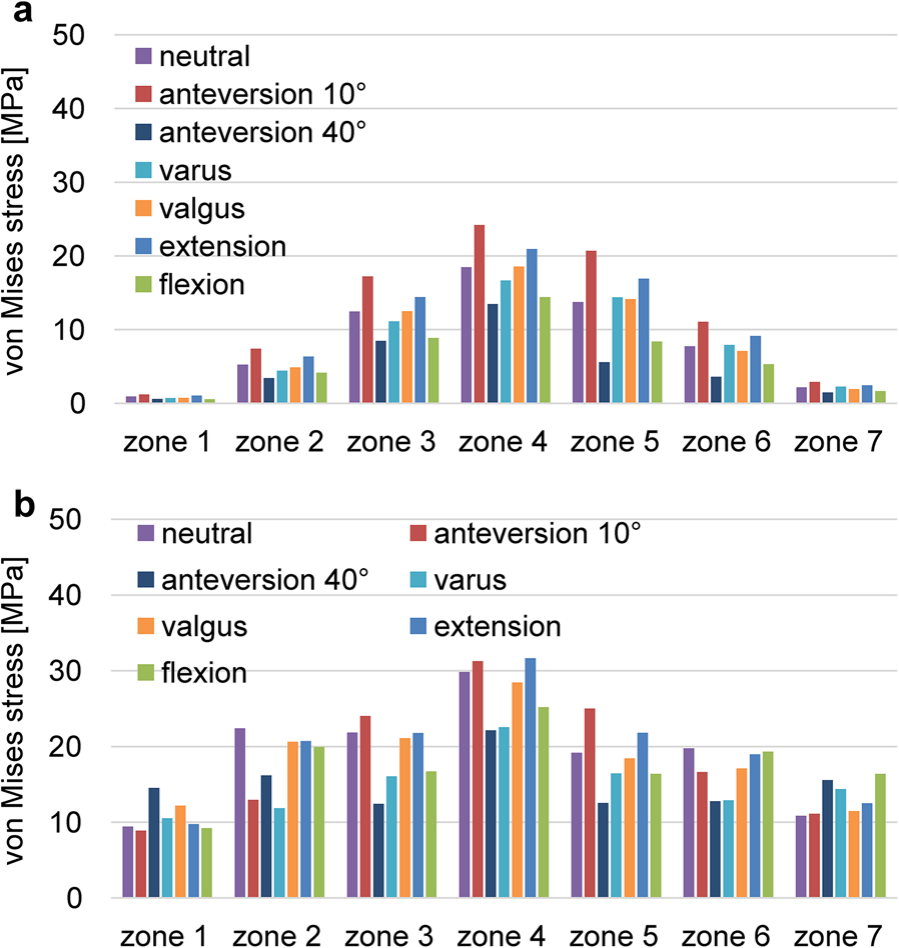
Comparisons of the average values of von Mises stress under the standing condition. **a** PROFEMUR® Preserve. **b** PROFEMUR® TL. The difference between the PROFEMUR® Preserve and PROFEMUR® TL is a greater von Mises stress in the standing position than in the walking condition. PROFEMUR® Preserve and PROFEMUR® TL are manufactured by MicroPort Orthopedics (Arlington, TN, USA)

**Fig. 7:**
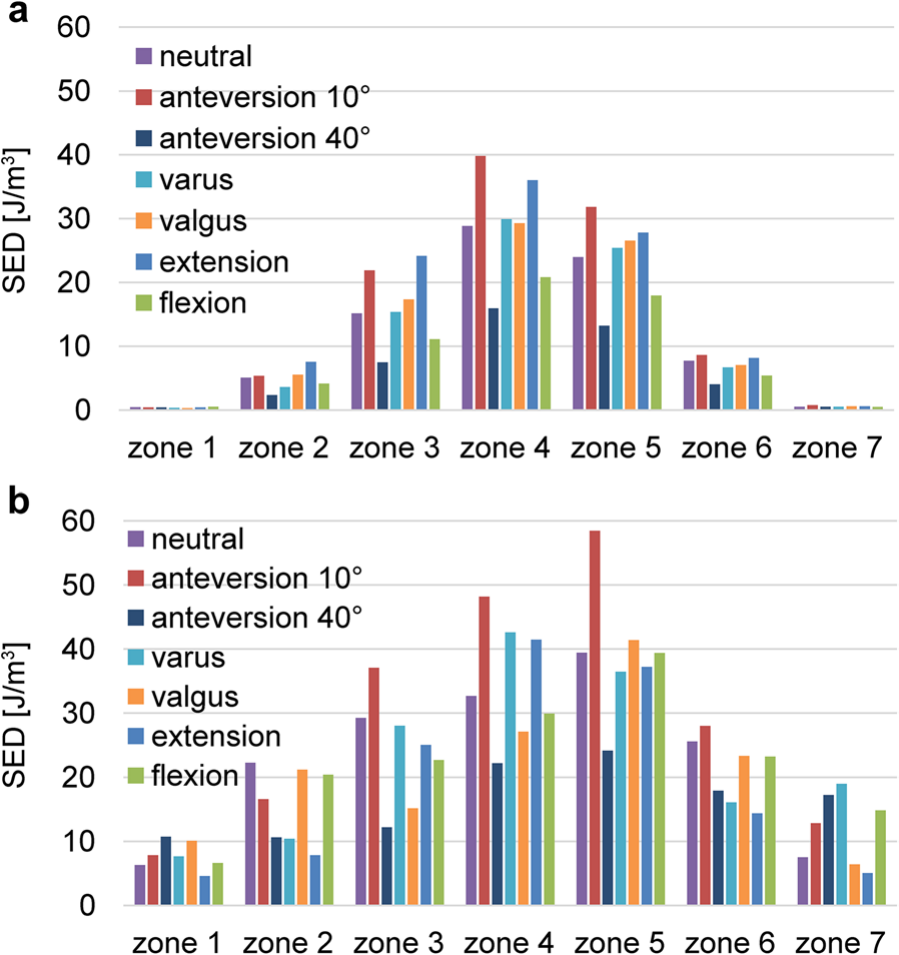
Comparisons of the average values of strain energy density under the walking condition. **a** PROFEMUR® Preserve. **b** PROFEMUR® TL. The strain energy density during walking is nearly the same as the von Mises stress findings during walking. Those of zones 1 and 7 in the PROFEMUR® Reserve are very small. PROFEMUR® Preserve and PROFEMUR® TL are manufactured by MicroPort Orthopedics (Arlington, TN, USA)

**Fig. 8:**
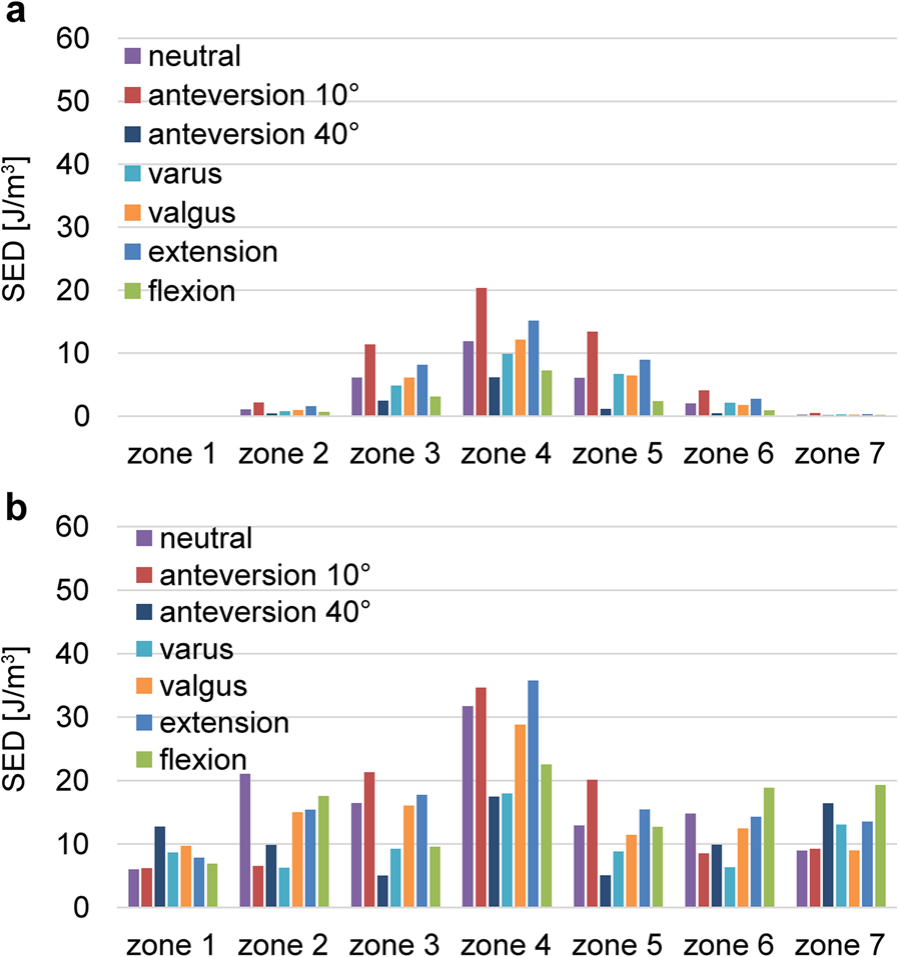
Comparisons of the average values of strain energy density under the standing condition. **a** PROFEMUR® Preserve. **b** PROFEMUR® TL. The difference between PROFEMUR® Preserve and PROFEMUR® TL is larger in strain energy density during standing than during walking, which is similar to the von Mises stress results. PROFEMUR® Preserve and PROFEMUR® TL are manufactured by MicroPort Orthopedics (Arlington, TN, USA)

The stress cloud map of von Mises stress distribution of femur models with neutral stem positions under the walking condition is shown in Fig. 4. As shown in Figs. 5 and 7 under the walking condition and Figs. 6 and 8 under the standing condition, the PROFEMUR® Preserve stem exhibited mountainous von Mises stress and SED distribution with the apex at zone 4, and the PROFEMUR® TL exhibited a mountainous von Mises stress and SED distribution on the apex at zone 4 or zone 5. The PROFEMUR® TL had a steeper mountain shape than did the PROFEMUR® Preserve. The difference between the PROFEMUR® Preserve and the PROFEMUR® TL was greater in the standing position than in the walking position, as shown in Figs. 5, 6, 7, and 8.

As shown in Figs. 5 and 6, the PROFEMUR® TL showed an increase in von Mises stress in zone 7 with the varus position, but the PROFEMUR® Preserve did not show any increase in Mises stress in zone 7, even in the varus position. In the PROFEMUR® Preserve, as shown in Tables 6 and 7, the von Mises stress generated around the stem was greater in the extended position than in the flexed position. As shown in Tables 6 and 7 and Figs. 5 and 6, the von Mises stress generated around the stem tended to be lower with a larger stem anteversion than with a smaller stem anteversion. Even though stem anteversion findings differed, the PROFEMUR® Preserve had a lower von Mises stress than did the PROFEMUR® TL in zones 1 and 7.

**Table 6:** Average values of von Mises stress and strain energy density under the walking condition

	Zone 1		Zone 2		Zone 3		Zone 4		Zone 5		Zone 6		Zone 7	
	Preserve	TL	Preserve	TL	Preserve	TL	Preserve	TL	Preserve	TL	Preserve	TL	Preserve	TL
*von Mises stress (MPa)*														
Neutral	0.92	9.41	5.23	22.38	12.48	21.84	18.48	29.81	13.74	19.17	7.74	19.78	2.16	10.85
Varus	0.70	10.53	4.42	11.84	11.13	16.05	16.67	22.52	14.39	16.45	7.94	12.87	2.24	14.38
Valgus	0.73	12.19	4.86	20.61	12.50	21.09	18.57	28.43	14.13	18.42	7.12	17.10	1.94	11.46
Flexion	0.57	9.21	4.14	19.95	8.85	16.70	14.40	25.20	8.39	16.39	5.32	19.32	1.65	16.40
Extension	1.06	9.75	6.34	20.71	14.41	21.77	20.94	31.63	16.93	21.81	9.16	18.96	2.46	12.51
Anteversion 10°	1.20	8.87	7.43	12.97	17.22	24.03	24.19	31.24	20.69	24.99	11.08	16.63	2.91	11.12
Anteversion 40°	0.59	14.53	3.43	16.18	8.48	12.40	13.47	22.11	5.60	12.54	3.59	12.76	1.49	15.55
*SED (J/m*^*3*^*)*														
Neutral	0.10	5.98	1.06	21.04	6.09	16.44	11.87	31.72	6.08	12.92	2.02	14.78	0.24	8.95
Varus	0.04	8.67	0.75	6.26	4.83	9.22	9.87	17.96	6.69	8.79	2.10	6.33	0.27	13.06
Valgus	0.04	9.72	0.94	15.02	6.11	16.03	12.15	28.80	6.43	11.41	1.75	12.45	0.22	9.00
Flexion	0.04	6.87	0.66	17.57	3.06	9.56	7.22	22.54	2.38	12.67	0.92	18.84	0.19	19.29
Extension	0.10	7.84	1.55	15.37	8.12	17.74	15.15	35.74	8.96	15.44	2.74	14.28	0.29	13.54
Anteversion 10°	0.11	6.20	2.14	6.51	11.36	21.31	20.32	34.62	13.42	20.12	4.08	8.54	0.48	9.21
Anteversion 40°	0.04	6.87	0.43	17.57	2.45	9.56	6.12	22.54	1.10	12.67	0.44	18.84	0.15	19.29

*MPa* mega-pascal, *SED* strain energy density, *J/m*^*3*^ joule per cubic meter

**Table 7:** Average values of von Mises stress and strain energy density under the standing condition

	Zone 1		Zone 2		Zone 3		Zone 4		Zone 5		Zone 6		Zone 7	
	Preserve	TL	Preserve	TL	Preserve	TL	Preserve	TL	Preserve	TL	Preserve	TL	Preserve	TL
*von Mises stress (MPa)*														
Neutral	0.92	9.41	5.23	22.38	12.48	21.84	18.48	29.81	13.74	19.17	7.74	19.78	2.16	10.85
Varus	0.70	10.53	4.42	11.84	11.13	16.05	16.67	22.52	14.39	16.45	7.94	12.87	2.24	14.38
Valgus	0.73	12.19	4.86	20.61	12.50	21.09	18.57	28.43	14.13	18.42	7.12	17.10	1.94	11.46
Flexion	0.57	9.21	4.14	19.95	8.85	16.70	14.40	25.20	8.39	16.39	5.32	19.32	1.65	16.40
Extension	1.06	9.75	6.34	20.71	14.41	21.77	20.94	31.63	16.93	21.81	9.16	18.96	2.46	12.51
Anteversion 10°	1.20	8.87	7.43	12.97	17.22	24.03	24.19	31.24	20.69	24.99	11.08	16.63	2.91	11.12
Anteversion 40°	0.59	14.53	3.43	16.18	8.48	12.40	13.47	22.11	5.60	12.54	3.59	12.76	1.49	15.55
*SED (J/m*^*3*^*)*														
Neutral	0.10	5.98	1.06	21.04	6.09	16.44	11.87	31.72	6.08	12.92	2.02	14.78	0.24	8.95
Varus	0.04	8.67	0.75	6.26	4.83	9.22	9.87	17.96	6.69	8.79	2.10	6.33	0.27	13.06
Valgus	0.04	9.72	0.94	15.02	6.11	16.03	12.15	28.80	6.43	11.41	1.75	12.45	0.22	9.00
Flexion	0.04	6.87	0.66	17.57	3.06	9.56	7.22	22.54	2.38	12.67	0.92	18.84	0.19	19.29
Extension	0.10	7.84	1.55	15.37	8.12	17.74	15.15	35.74	8.96	15.44	2.74	14.28	0.29	13.54
Anteversion 10°	0.11	6.20	2.14	6.51	11.36	21.31	20.32	34.62	13.42	20.12	4.08	8.54	0.48	9.21
Anteversion 40°	0.04	6.87	0.43	17.57	2.45	9.56	6.12	22.54	1.10	12.67	0.44	18.84	0.15	19.29

*MPa* mega-pascal, *SED* strain energy density, *J/m*^*3*^ joule per cubic meter

## Discussion

Our results indicated that a short stem can reduce the stress load on the surrounding bone, compared to a tapered wedge. It can also reduce the effect of the varus position such as biomechanical imbalance or inferiority. Owing to stem alignment, the effect of varus and valgus position was small in the coronal plane; however, the stress generated around the stem was larger in the extended position than in the flexed position in the sagittal plane and the smaller stem anteversion in the horizontal plane.

The actual changes in the strain and stress generated in the femur by changing the stem length remain unclear. The longer the stem, the greater is the strain distal to the femur, and stress shielding proximally is more likely to be produced [2]. Reimerringer et al. [25] used finite element analysis to investigate the effect of stem length and design on initial fixation in cementless stems, and they showed that the amount of micromotion increased with decreasing stem length. By using mechanical experiments, Bieger et al. [26] reported that, compared to conventional stems, short stems slightly increase femoral strain proximal to the femur and the amount of subsidence; however, they maintained axial stability. Kwak et al. [16] used FEM analysis to show that the shorter stem increased micromotion between the bone and stem. In our study, by using a short stem with a relatively high medullary cavity occupancy, the stress distribution was nearly the same as that of the tapered wedge stem, but the stress and SED on the surrounding bone were smaller than those of the tapered wedge. A short stem with a short length has a smaller bending moment owing to the load acting on the stem head. Therefore, deformation of the bone around the stem is expected to be small, and the risk of fracture after installing the stem is considered low. Additionally, the SED is associated with bone remodeling [27]. In this respect, the very low SED of the PROFEMUR® Preserve in zones 1 and 7 raises concerns about bone resorption and weak bone ingrowth. Furthermore, the PROFEMUR® TL seems to be less prone to stress shielding than the PROFEMUR® Preserve because of its uneven stress distribution. The short stem is likely to have little effect on the actual range of stress shielding with less width of the zone. The results of this study demonstrated that the stresses applied to the surrounding area are different and possibly depend on the short stem design. This finding is important for the future designs of short stems.

Additionally, inserting a short stem in the exact intermediate position is difficult in terms of the surgical technique, and the short stem is prone to an incorrect alignment [5]. At a mean postoperative evaluation of 4 years, no significant differences existed in radiological and clinical evaluation, including stem subsidence between short stem placement in the varus and intermediate positions [28]. However, one study [29] using FEM analysis showed that varus placement increased strain stresses in the cortical bone around the short stem at the calcar and lateral to the distal tip. Kwak et al. [16] used FEM analysis to show that varus placement increased micromotions between the bone and stem more than did the intermediate placement. Simulations were conducted with a clinically possible 3° of varus placement in this study; however, no increase in stress occurred at the zone 7 calcar with the PROFEMUR® Preserve, compared to that with the PROFEMUR® TL. The results of this study may be associated with the matching of the stem design and medullary cavity shape of the femoral proximal. Therefore, even though a short stem should be inserted with an appropriate alignment, a wide mechanical safety zone exists that depends on the short stem design.

**Fig. 9:**
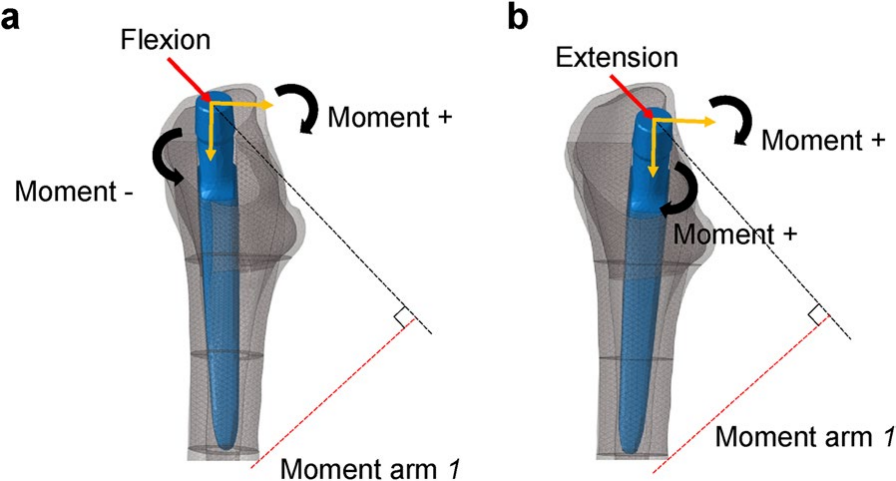
Comparison of moment arm between models with flexion and extension stem positions. **a** Flexion. **b** Extension. The moment is larger in extension than in flexion because of the longer moment arm l. The moment due to the downward load component in flexion is working in the opposite direction; therefore, the composite moment is smaller

**Fig. 10:**
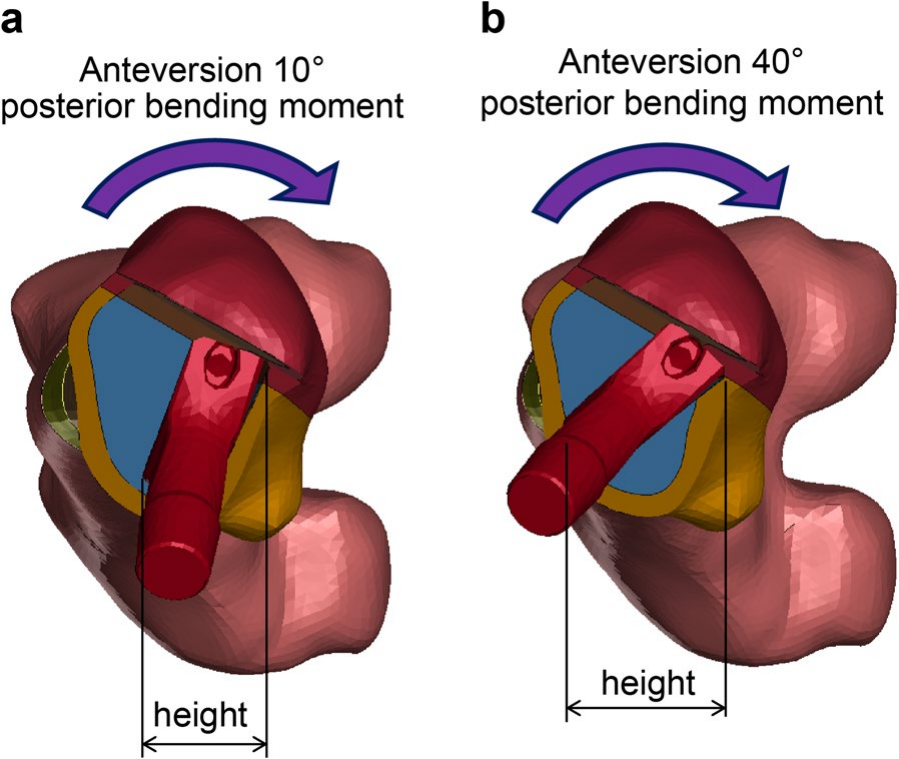
Comparison of the height of area moment of inertia between models in different stem anteversion. **a** Anteversion 10°. **b** Anteversion 40°. The bending stress is smaller for anteversion 40° than for anteversion 10° because the height corresponding to the height of the cross-sectional second moment is larger

To the best of our knowledge, our study is the first to report the effect of changes in alignment in the coronal, sagittal, and horizontal planes. The results showed that the stresses loaded on the surrounding bones tended to be higher for extension placement than for flexion in the sagittal plane and for 10° anteversion than for 40° anteversion in the horizontal plane. This difference in stress may be because of the difference in the magnitude of moment with hip loading. Comparing the flexion and extension installation of the PROFEMUR® Preserve, the bending moment is proportional to the length of the moment arm; therefore, a longer moment arm induced a larger bending moment. As shown in Fig. 9 of the sagittal plane, the bending moment is larger in extension than in flexion because the moment arm is longer. Another reason may be that the downward load component induces a forward bending moment during the flexion installation. However, these findings have not been confirmed for the PROFEMUR® TL. The stiffness of the femur with the longer stem length of the PROFEMUR® TL is higher than that of the PROFEMUR® Preserve. We speculate that this effect is greater than that of the moment arm and other factors previously mentioned. However, in the horizontal plane, the bending stress is inversely proportional to the cross-sectional secondary moment. Therefore, the larger the height, which corresponds to the height of the cross-sectional secondary moment, the larger is the cross-sectional secondary moment. The bending stress is also greatly reduced. Figure 10 shows that the height of the anteversion 40° is larger and the bending stress is smaller than that of the anteversion 10°. Owing to the aforementioned causes, different magnitudes of stress are generated around the stem, depending on the installation alignment.

A limitation of this study is that the stem geometry and the surface finish between the two compared stems are different. Therefore, our results may not reflect real world changes that occur because of the shorter stem. This study’s findings can be applied primarily in the early postoperative period rather than after osseointegration has been achieved. A single femur was used in this study; however, in practice, femurs vary in medullary cavity shapes with each bone quality. We used implant size in this study so that the leg length would not be altered, although the proximal end of the PROFEMUR® Preserve stem is 1 mm lateral to the PROFEMUR®TL. In this study, we could not evaluate for individual differences among patients such as weight, bone strength, femoral marrow cavity geometry or evaluate them, based on various femoral stems with customized design or stiffness, which were addressed in another study [30]. The loading conditions used in this study were for standing and walking. However, stresses could not be compared between the braking and propulsive phases of the walking cycle because only the loads at the time when ground reaction force reached the maximum value were used in the simulation. Stair climbing, which is a more severe loading condition, was not simulated at this time. Kwak et al. [16] used FEM analysis to show that the micromotion between the bone and stem increases when climbing stairs. In addition, the present study did not include conditions such as different stem designs, medullary geometries, inappropriate stem sizes, and bone strength factors such as bone density. Despite the limitations of our study, the results realistically reveal the specific effects and differences for existing stems with various alignments.

## Conclusion

Using an actual femoral stem, the von Mises stress, SED, and maximum principal stress of the surrounding bone were investigated by Gruen zone. The effect of stem alignment was also shown in three directions, based on seven conditions. In the design of stems used in this study, the trend of stress distribution was similar, regardless of the stem length. The PROFEMUR® Preserve with the short stem generated less stress around the stem than did the PROFEMUR® TL with a tapered wedge stem. In clinical practice, the initial fixation of the stem depends on factors, such as the bone strength of the femur, shape of the medullary cavity, and height of the stem placement. A surgeon must sufficiently understand the mechanical characteristics of a stem model, especially when using short stems, and install them successfully after considering the effects of changes in alignment on the surrounding stresses. For the development of superior stems and improvement in surgical techniques, more detailed conditioned studies are desirable, while considering the limitations of this study.

## Data Availability

The datasets generated and/or analyzed during the current study are available from the corresponding author upon reasonable request.

## Abbreviations

3D: Three-dimensional
CT: Computed tomography
FEM: Finite element method
SED: Strain energy density
THA: Total hip arthroplasty
VOI: Volume of interest
